# Shelf‐Life Extension of Chicken Fillets Using Flaxseed Mucilage Coatings Incorporated With Bixin‐Allicin or Bixin‐Orange Peel Essential Oil Nanoemulsions

**DOI:** 10.1002/fsn3.71428

**Published:** 2026-01-07

**Authors:** Neda Hashemi, Elnaz Milani, Arash Koocheki

**Affiliations:** ^1^ Center of Pardisan Hospitality and Tourism Management University of Applied Science and Technology Tehran Iran; ^2^ Department of Food Processing Iranian Academic Center for Education Culture and Research (ACECR), Khorasan Razavi Mashhad Iran; ^3^ Department of Food Science and Technology Ferdowsi University of Mashhad Mashhad Iran

**Keywords:** antimicrobial activity, antioxidant activity, chicken fillets, food preservation

## Abstract

This study evaluated the properties of orange peel essential oil (OPE) and allicin emulsions and their efficacy in flaxseed mucilage‐bixin (FM‐B) coating for preserving chicken fillets during 12‐day storage at 4°C. The nanoemulsions (OPEN and AN) exhibited superior initial properties and stability, with significantly smaller initial droplet sizes (93.5 and 81.3 nm, respectively), high zeta potential (−36.46 mV for both), and higher antioxidant activity (33.27 and 42.28 mg/mL) compared to the corresponding microemulsions (OPEM and AM). This enhanced stability was confirmed after 30 days of storage, as the nanoemulsions maintained their small droplet size (117 nm for OPEN and 105 nm for AN), while the microemulsions exhibited significantly larger droplets (1304 nm for OPEM and 1070 nm for AM) and a lower zeta potential (approximately −26.7 mV). Coated fillets demonstrated significantly improved preservation over the uncoated control (C). The best performance was consistently observed for the FM‐B‐AN and FM‐B‐OPEN coatings. After 12 days of storage, the FM‐B‐AN coating limited the pH increase to 6.29, weight loss to 7.17%, and maintained the fillet hardness at 4.53 N, compared to the control values of pH 7.30, 16.43% weight loss, and 3.44 N hardness. Lipid oxidation was significantly inhibited, with FM‐B‐AN producing the lowest peroxide value (PV) and thiobarbituric acid reactive substances (TBARs) of 4.90 meq O_2_/kg and 0.26 mg MDA/kg, respectively, versus 11.72 meq O_2_/kg and 0.77 mg MDA/kg for the uncoated sample. Microbiological analyses revealed that all active coatings inhibited the microbial growth, with FM‐B‐AN and FM‐B‐AM being the most effective coatings. On day 12, FM‐B‐AN achieved the lowest total viable bacterial count (TVC) (7.25 log CFU/g), psychrotrophic bacteria count (PTC) (6.90 log CFU/g), molds and yeasts (5.80 log CFU/g), *E. coli* (4.60 log CFU/g), and *S. aureus* (0.77 log CFU/g). In conclusion, the incorporation of allicin and OPE nanoemulsions, particularly allicin nanoemulsion (AN), into the FM‐B coating created a highly effective barrier that delayed the physicochemical deterioration and microbial growth in chicken fillets.

## Graphical abstract

Flaxseed mucilage incorporated with bixin‐allicin or bixin‐orange peel oil nanoemulsions significantly extended the shelf‐life and quality of fresh chicken fillets.

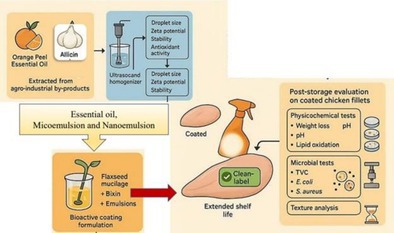

## 1 Introduction

Poultry meat has emerged as the leading choice in global meat production, representing nearly half of total meat consumption due to its cost‐effectiveness and sustainable production (FAO 2023). According to FAO (2023), global poultry meat production reached 140 million tons in 2023, with chicken being the most consumed type owing to its high protein content and favorable low‐fat levels. However, throughout the supply chain from slaughter and transportation to storage and preparation, chicken meat remains susceptible to microbial contamination (Lyu et al. 2024). Furthermore, its high moisture and protein content make it particularly prone to spoilage by microorganisms (Zhu et al. 2022). To address these challenges, interventions such as hygienic slaughter and processing, cold chain management, antimicrobial packaging, and natural preservatives are used to extend shelf life and ensure safety (Fabio et al. 2025; Lyu et al. 2024).

Extensive research has been conducted on multifunctional packaging compounds specifically designed to maintain poultry meat quality (Gautam et al. 2023; Gil and Rudy 2023; Peter et al. 2023). Active coatings are edible suspensions applied to food surfaces to form a thin, transparent layer upon drying. These coatings are increasingly used in the food industry due to their ability to act as effective barriers against water vapor, oxygen, and carbon dioxide. Additionally, they can serve as carriers for antimicrobial agents, enhancing food preservation (Tripathi et al. 2021). A key advantage of edible coatings is their ability to enhance the appearance of food products by providing a glossy finish (Perez‐Vazquez et al. 2023; Tripathi et al. 2021). In response to the environmental challenges posed by synthetic packaging, researchers have focused on developing biopolymer‐based alternatives. Among these, polysaccharides including starches, mucilages, and gums have emerged as promising materials for edible coating applications (Nunes et al. 2023; Perez‐Vazquez et al. 2023). Flaxseed mucilage‐based edible coatings offer excellent water‐holding capacity, emulsifying activity, and film‐forming ability (Tee et al. 2016). Due to their inherent antimicrobial and antioxidant properties, these coatings can effectively extend the shelf life of perishable foods by preventing oxidative degradation and microbial spoilage (Manzoor et al. 2023). However, their widespread assumption requires overcoming certain limitations, particularly in terms of thickness, mechanical strength, barrier properties (water and oxygen resistance), and desirable physical characteristics such as lightness, flexibility, and transparency (Salehi 2020; Tripathi et al. 2021).

Essential oils exhibit significant antimicrobial properties that effectively enhance food safety and extend the shelf life of perishable products (Wang et al. 2021; Zhang et al. 2022). Researches have demonstrated the potential of orange peel waste‐derived essential oil (OPE) as a sustainable natural additive for food applications, particularly in bio‐based edible coatings and packaging (Giello et al. 2024). Due to its significant antimicrobial and antioxidant properties, along with its pleasant aroma, this extract shows promise for extending the shelf life and enhancing the microbial safety of perishable food products (Cebi et al. 2020; Feng 2022; Golmohammadi et al. 2018; Shaw et al. 2023). The primary bioactive components of OPE are monoterpenes (particularly limonene, which constitutes 85%–96% of the oil) and their oxygenated derivatives. Limonene exhibits notable antibacterial and antiviral activities, making it particularly valuable for food preservation and therapeutic applications (Lagha‐Benamrouche and Madani 2013; Park et al. 2020). Allicin (allyl 2‐propenethiosulfinate), the primary sulfur‐containing compound in garlic extract, has been successfully incorporated into various edible coatings and films to enhance their antimicrobial functionality. Researches have shown that coatings containing allicin exhibited bacteriostatic activity against foodborne pathogens such as *E. coli* and *S. aureus* , while simultaneously preserving food quality through its antioxidant activity (Hassanzadeh et al. 2023; Li et al. 2021; Salehi 2020). These dual functional properties enable allicin‐based coatings to effectively delay the microbial spoilage and lipid oxidation in perishable foods, thereby extending the final shelf life of the product (Li et al. 2021).

Essential oil nanoemulsions serve as effective carrier systems that enhance the stability, solubility, and antimicrobial efficacy of essential oils (Islam et al. 2023; Wang et al. 2021; Zaharioudakis et al. 2023). Compared to essential oil macroemulsions, nanoemulsions incorporated into coatings provide superior protection against environmental factors such as light, oxygen, and degradation, while minimizing evaporation during the coating drying process (Qiu et al. 2022). Additionally, essential oil nanoemulsions improve the physical and mechanical properties of emulsion coatings, including improved uniformity, adhesion, tensile strength, and enhanced barrier properties against moisture and gases (Medeleanu et al. 2023; Wang et al. 2021; Zhao et al. 2023). Several studies have demonstrated the effectiveness of essential oil‐based nanoemulsions in meat preservation. Lemon myrtle essential oil nanoemulsions have shown potential for extending the shelf life of monkey meat (Zixiang et al. 2021), while Plantago seed mucilage incorporated with lemon essential oil improved buffalo fillet preservation (Noshad et al. 2021). Similarly, *Myristica fragrans* (nutmeg) essential oil nanoemulsions enhanced beef quality (Kiarsi et al. 2020). Furthuremore, nanoemulsions formulated with grape and cinnamon essential oils enhanced the preservation of mullet fillets (Ameur et al. 2022). These findings confirm that bioactive compound‐loaded nanoemulsions can significantly improve the microbiological, physicochemical, and sensory attributes of fresh muscle foods, thereby extending their shelf life.

Annatto seeds impart an orange‐to‐red hue due to their primary carotenoids, oil‐soluble bixin and water‐soluble norbixin (Chisté et al. 2011; Van Chuyen et al. 2012). Due to its lipophilic nature, bixin exhibits excellent compatibility with lipid‐based systems such as nanoemulsions and fat‐rich edible coatings, thereby enhancing its dispersion stability and overall performance (dos Coelho Santos et al. 2022). Besides its coloring properties, bixin demonstrates antimicrobial and antioxidant activity, making it particularly valuable for functional food applications (Ashraf et al. 2023; dos Coelho Santos et al. 2022; Martín‐Sánchez et al. 2014). Bixin can be effectively incorporated into various biopolymer matrices to create active food packaging that inhibits oxidative degradation. For instance, bixin‐based coatings have been shown to delay the fruit ripening and senescence (Oliveira et al. 2025), slow the increase of peroxide values in oils, and protect sensitive nutrients (Stoll et al. 2023). Its efficacy is retained even when nanoencapsulated in starch films, underscoring its broad applicability for maintaining food quality and safety (Pagno et al. 2016). This superior performance is attributed to bixin's unique molecular structure, which optimizes its release and barrier properties within polymer matrices such as polylactic acid, highlighting its efficacy as a natural additive for preserving lipid‐based foods (Stoll et al. 2019).

While flaxseed mucilage has emerged as a promising biodegradable polymer for coating formation, its potential as a matrix for bixin is largely unexplored. Furthermore, a significant research gap exists concerning the potential synergistic effects between bixin and natural antimicrobials such as OPE or allicin when within a coating system. Therefore, this study aims to investigate, for the first time, the efficacy of a novel flaxseed mucilage‐based coating incorporating bixin in combination with either OPE or allicin nanoemulsions to enhance the shelf‐life and quality of refrigerated chicken fillets. The study comprehensively evaluated the preservative efficacy of the developed coatings by monitoring specific microbial indicators (e.g., total viable count, *Staphylococcus aureus* , *E. coli* , molds and yeasts), physicochemical parameters (pH, weight loss), and lipid oxidation markers (peroxide value and TBARS) during refrigerated storage. This approach offers a promising natural strategy for extending the shelf life of poultry products while maintaining their safety and quality.

## 2 Methodes and Materials

### 2.1 Materials

Annatto seeds were procured from Hyderabad, India and stored at −18°C in light‐protected polyethylene bags until use. All chemical reagents used for analysis were of analytical grade and obtained from Merck (Darmstadt, Germany). Microbial culture media were sourced from Sigma‐Aldrich (Munich, Germany), while allicin (≥ 98% purity) was purchased from NutriHerb BioTech Co. Ltd. (Xi'an, China).

### 2.2 Extraction of Bixin From Annatto Seed

Bixin was extracted following the method of Van Chuyen et al. (2012) with slight modifications. Semi‐defatted annatto seeds (20 g) were placed in 250 mL amber flasks, wrapped in aluminum foil to prevent light degradation, and mixed with chloroform at a 1:12.9 (w/v) seed‐to‐solvent ratio. The sealed flasks were incubated at 48°C with constant shaking (250 rpm) for 120 min. After extraction, the mixture was vacuum filtered through Whatman No. 1 filter paper (15 μm pore size). Chloroform was removed using a rotary evaporator at 35°C under light‐protected conditions, and the remaining pigment was dried to constant weight in a desiccator. The final extract was stored at −18°C in light‐protected containers.

### 2.3 Extraction of Orange Peel Essential Oil (OPE)

Fresh orange peels were collected from local juice shops (Mashhad, Iran), dried at 45°C for 24 h, and milled. Essential oil extraction was performed using Shaw et al. (2023) method under optimized conditions: solid to liquid ratio of 2:3 (w/v), extraction time of 120 min, and temperature of 100°C. The obtained orange peel essential oil (OPE) was transferred to amber glass vials and stored at −18°C until further analysis.

The chemical composition of the essential oil was analyzed using an Agilent 7890A GC–MS system (Agilent Technologies, USA) equipped with an HP‐5MS capillary column (30 m × 0.25 mm i.d. × 0.25 μm film thickness; 5% phenylmethylsiloxane stationary phase). The thermal program ranged from 50°C to 270°C with a temperature rise of 3°C/min. The injector and transfer line temperatures were maintained at 280°C and 290°C, respectively. High purity helium was used as the carrier gas at a constant flow rate of 1.0 mL/min. Electron impact ionization was performed at 70 eV. Compound identification was achieved by comparing retention indices and mass spectra with reference standards from the Wiley GC/MS Library (version NIST 14), Mainlib, and Replib databases (Noori et al. 2018).

Functional group analysis was performed using a Thermo Nicolet AVATAR‐370 FTIR spectrometer (Thermo Fisher Scientific, USA). The OPE sample was mixed with spectroscopic‐grade potassium bromide (1:100 ratio) and pressed into a transparent pellet. Infrared spectra were recorded in the 400–4000 cm^−1^ range with 32 scans at 4 cm^−1^ resolution (Cebi et al. 2020).

### 2.4 Flaxseed Mucilage Extraction

Flaxseed oil cakes, a by‐product from cold‐pressed oil extraction in Mashhad, Iran, were air‐dried, milled, and sieved (60 mesh) to obtain a fine powder and then stored under cool and dry conditions. For mucilage extraction, 30 g of powder was mixed with 900 mL distilled water (1:30 w/v) and stirred at 1000 rpm using a magnetic hotplate stirrer (LMS Co. Ltd., HTS‐1003, Tokyo, Japan) while heated at 80°C–100°C for 3 h. The mixture was then cooled to 25°C, centrifuged at 3900 rpm for 15 min using a centrifuge (Hettich Lab Technology, Universal 320R, USA), and the remaining mucilage was recovered by filtering the seeds through cheesecloth (Tee et al. 2016).

### 2.5 Emulsion Properties

#### 2.5.1 Preparation of Micoemulsion and Nanoemulsion

The preparation of bixin/OPE microemulsion (B‐OPEM) and nanoemulsion (B‐OPEN), along with bixin/allicin microemulsion (B‐AM) and nanoemulsion (B‐AN), was conducted following the method described by Hassanzadeh et al. (2023) with slight modifications. The process began by mixing 0.1 g of bixin with each oil phase (10%) and Tween 80 (10%) in a 1:1 oil‐to‐surfactant ratio. The mixture was then homogenized at room temperature using a magnetic stirrer (IKA T25 basic, Germany) at 500 rpm for 15 min to ensure uniform dispersion. An aqueous phase consisting of 55% distilled water and 25% glycerol was then gradually added dropwise to the oil phase while maintaining continuous stirring at 500 rpm for an additional 30 min.

For microemulsion preparation, the mixture was homogenized using a VIRTIS TEMPEST homogenizer (model 302,968) at 11,000 rpm for 10 min. Nanoemulsions were then produced by subjecting the pre‐homogenized emulsion to ultrasonic processing (Bandelin, Germany) at 500 W output power using a 20 mm probe diameter at 40% amplitude and 20 kHz frequency for 15 min. Throughout sonication, the temperature was maintained at 25°C ± 1°C through a water‐cooled jacket to prevent thermal degradation of heat‐sensitive components.

#### 2.5.2 Particle Size and Zeta Potential

The hydrodynamic diameter (Z‐average), polydispersity index (PDI), and zeta potential of the essential oil micro and nanoemulsions were determined in triplicate by dynamic light scattering (Zetasizer Nano ZS90, Malvern Instruments, UK) at 25°C. Results represent the mean droplet size distribution of three independent measurements.

#### 2.5.3 Emulsion Stability

To determine the stability of each emulsion, samples were stored in the refrigerator for 1 month, and their particle sizes were measured by a DLS device every week (Chu et al. 2020).

#### 2.5.4 Antioxidant Activity (DPPH)

The antioxidant activity of the emulsions was determined based on their ability to scavenge the stable DPPH (2,2‐diphenyl‐1‐picrylhydrazyl) free radical, following a modified method described by Kiarsi et al. (2020). Briefly, 50 μL of the emulsion sample (diluted in methanol at concentrations of 100–400 μg/mL) was added to 5.0 mL of a 0.2 mM DPPH solution prepared in methanol. The mixture was incubated in the dark at room temperature for 30 min. The absorbance was then measured at 517 nm using a UV–Vis spectrophotometer. A control sample was prepared by replacing the emulsion with methanol. The radical scavenging activity (RSA) was calculated using the following equation:

(1) $$\begin{array}{c} \text{DPPH inhibition activity}=\left(A\;\text{control}—A\;\text{sample}/A\;\text{control}\right)\\ \times 100 \end{array}$$

### 2.6 Preparation of Edible Coating

To prepare the edible coating, 2 g of FM and 0.5 g of Tween 80 were dissolved in 100 mL of distilled water. The mixture was then homogenized at 50°C using a magnetic stirrer (500 rpm, 30 min) and refrigerated at 4°C for 24 h to ensure complete hydration. Antimicrobial emulsions were individually incorporated into the coating solution at 1% (w/v). Each formulation was homogenized (500 rpm, 10 min) and sterilized under UV light (1 h) according to the method of Heydari et al. (2020).

### 2.7 Coating the Chicken Fillets

Fresh chicken fillets were aseptically cut into 2 × 2 × 2 cm cubes and divided into seven groups: control (C, uncoated), FM (flaxseed mucilage), FM‐B (Flaxseed Mucilage + Bixin), FM‐B‐OPEM (Flaxseed Mucilage + Bixin + Orange Peel essential Oil Microemulsion), FM‐B‐AM (Flaxseed Mucilage + Bixin + Allicin Microemulsion), FM‐B‐OPEN (Flaxseed Mucilage + Bixin + Orange Peel essential Oil Nanoemulsion), and FM‐B‐AN (Flaxseed Mucilage + Bixin + Allicin Nanoemulsion). Each group was dip‐coated twice (30 s per immersion), drained on sterile sieves (5 min), and stored in pre‐sterilized polystyrene containers at 4°C ± 1°C (Noori et al. 2018). Microbiological and physicochemical analyses were conducted at 0, 4, 8, and 12 days.

### 2.8 Microbiological Properties of Chicken Fillets

For microbiological analysis, 5 g of coated chicken fillet samples were homogenized with 45 mL of 0.1% peptone water (250 rpm, 1 min), serially diluted (10^−1^–10^−8^), and plated onto selective media: Plate Count Agar (PCA) for total viable count (TVC, 37°C/24 h) and psychrotrophic bacteria (PTC, 7°C/10 days), Sabouraud Dextrose Agar (SDA) for mold/yeast (27°C/72 h), Eosin Methylene Blue (EMB) for *E. coli* (37°C/24 h), and Mannitol Salt Agar (MSA) for *S. aureus* (37°C/24 h). After incubation, live cells were counted, and the results were presented as log CFU/g (Kiarsi et al. 2020).

### 2.9 Physiccochemical Properties of Chicken Fillet

#### 2.9.1 pH

A 10 g sample of chicken fillet was homogenized with 90 mL of distilled water using a homogenizer at 10,000 rpm for 30 s. The pH of the homogenate was measured at 25°C using a calibrated pH meter (STARTER 3000, OHAUS, Switzerland). All measurements were performed in triplicate.

#### 2.9.2 Texture

Sample hardness was measured using a texture analyzer (TA‐Plus, AMETEK Lloyd Instruments, USA) equipped with a 36 mm cylindrical probe. The instrument, operating at a crosshead speed of 5 mm/s, applied a 10 kg compression force to 2 × 2 × 2 cm samples. Hardness was recorded as the peak compression force (N) required to deform each sample (Heydari et al. 2020). All measurements were performed in triplicate at ambient temperature (25°C ± 1°C).

#### 2.9.3 Weight Loss

The weight loss (WL%) of the samples was determined by recording the fillet weights with a precision balance (accuracy ±0.01 mg) throughout the storage period. The percentage of weight loss was calculated using the following equation (Garavito et al. 2020):

(2) $$\mathrm{WL}\;\left(\%\right)=\frac{\left(W_{\mathrm{ini}}-W_{t}\right)}{W_{\mathrm{ini}}}\times 100$$

where *W*_ini_ represents the initial weight and *W*_*t*_ is the sample weight at time *t*.

#### 2.9.4 Lipid Oxidation

Lipid oxidation was monitored throughout refrigerated storage by quantifying primary and secondary oxidation products. Peroxide value (PV) and thiobarbituric acid reactive substances (TBARS) were determined, with PV representing hydroperoxides (primary oxidation products) and TBARS measuring malondialdehyde equivalents (secondary oxidation products) in the coated chicken fillets. The PV was measured according to the method described by Bazargani‐Gilani et al. (2015). A 0.30 g sample was mixed with 9.8 mL of a chloroform‐methanol solution in a glass tube and vortexed briefly (2–4 s). Subsequently, 0.05 mL of 10 mM ammonium thiocyanate solution was added, followed by 0.05 mL of iron (II) solution, with brief vortexing after each addition. The mixture was then incubated at room temperature for 5 min, and the absorbance was measured at 500 nm using a UV–visible spectrophotometer. The PV was expressed as milliequivalents of O_2_/kg of fat.

The TBARS were measured based on the following method (Bazargani‐Gilani et al. 2015). A 10 g sample portion was homogenized with 1 mL of butylated hydroxytoluene (BHT, 1 mg/mL) and 35 mL of 5% trichloroacetic acid (TCA) solution. The homogenate was transferred to a distillation flask, diluted with 100 mL of distilled water, and subjected to distillation. The first 50 mL of distillate was collected and filtered through a Whatman No. 1 filter paper. Subsequently, 5 mL of the filtrate was mixed with 5 mL of 0.02 M thiobarbituric acid (TBA) solution and heated in a water bath at 100°C for 60 min. After cooling to room temperature, the absorbance was measured at 532 nm using a blank of water. The TBARS value was calculated from a standard curve prepared using 1,1,3,3‐tetraethoxypropane (TEP) and expressed as milligrams of malondialdehyde equivalents per kilogram of sample (mg MDA/kg).

### 2.10 Statistical Analysis

The experimental data were analyzed using a completely randomized factorial design in Minitab 18.0 (Minitab Inc., State College, PA, USA). Treatment means were compared against the control group using Dunnett's test and pairwise *t*‐tests at a 95% confidence level (*α* = 0.05). All analyses were performed in triplicate, with results considered statistically significant at *p* < 0.05.

## 3 Results and Discussion

### 3.1 Chemical Structure and Total Phenolic Content of OPE

Orange peel essential oil (OPE) was found to contain 27 compounds, representing 99.8% of the total essential oil, as shown in Table 1. The primary component of the oil was the monoterpene hydrocarbon (D‐limonene), which accounted for 86.1% of the composition. Other compounds identified included gamma‐terpinene, sabinene, alpha‐pinene, decanal, and alpha‐terpinene. These findings align with the study by Park et al. (2020), who reported that D‐limonene content in citrus peel essential oils ranged from 85.5% to 93.1%. Variations in the quantity and composition of compounds extracted from OPE can be attributed to factors such as fruit age and maturity, soil composition, genetics, climate, and extraction methods. According to Golmohammadi et al. (2018), limonene content in OPE extracted using water and steam was 77.39% and 89.13%, respectively.

**TABLE 1:** Chemical compositions of orange peel essential oil.

Component name	%	Component name	%
D‐Limonene	86.1	β‐Cubebene	0.13
Octanal	2.36	Valencene	0.11
β‐Myrcene	2.34	α‐Terpinolene	0.11
Linalool	2.21	β‐Pinene	0.08
Sabinene	1.21	β‐Cadinene	0.08
α‐Pinene	1.06	α‐Farnesene	0.04
Decanal	0.85	Phenol	0.03
α‐Cubebene	0.50	trans‐β‐Farnesene	0.02
Delta‐3‐Carene	0.42	Camphene	0.01
β‐Phellandrene	0.33	Cathine	0.01
2,6‐Octadiena	0.28	Phenethylamine	0.01
Phenylpropanolamine	0.25	Acetamide	0.01
γ‐Terpinene	0.21	Citronellal	0.16

**FIGURE 1:**
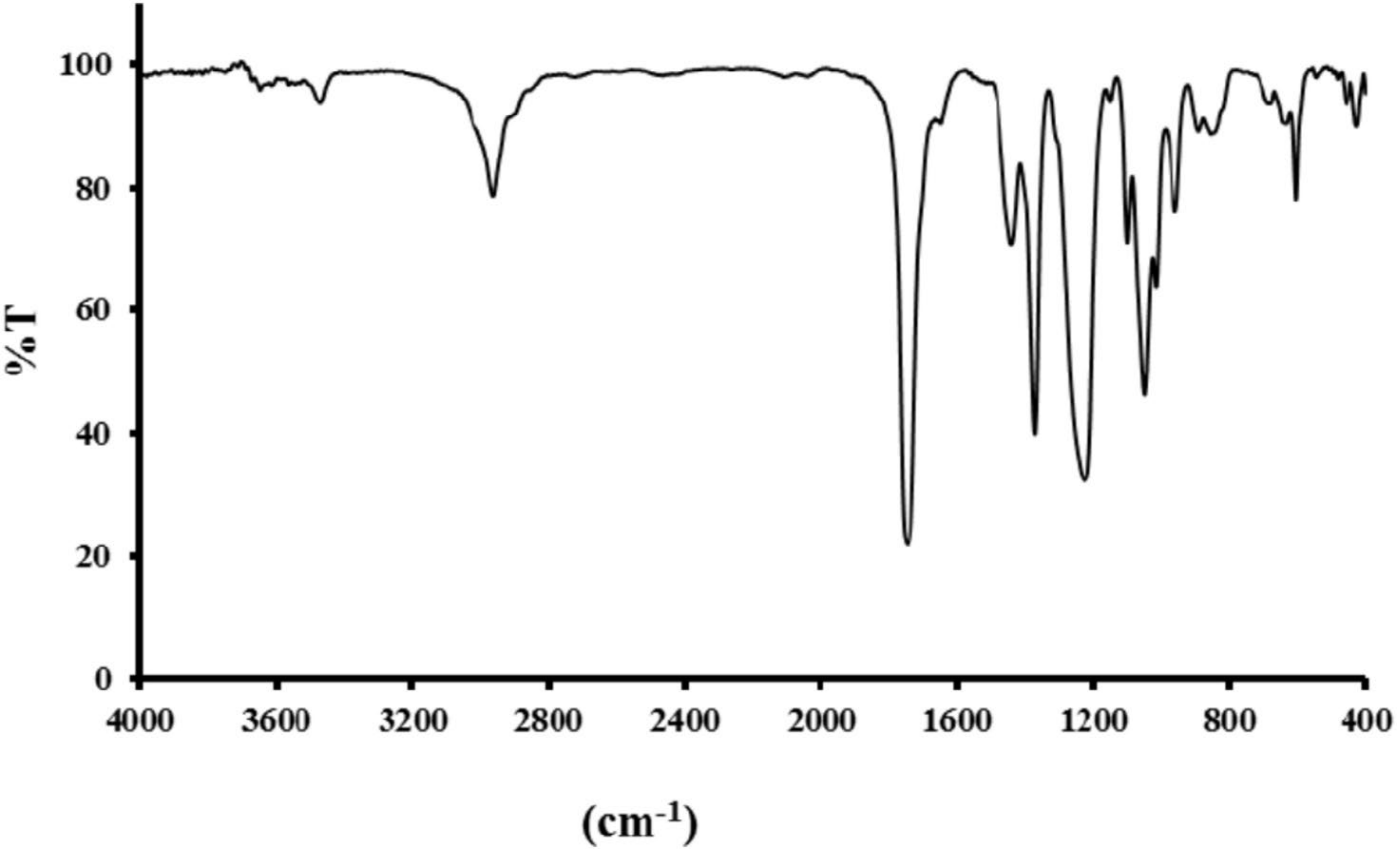
FTIR spectrum of orange peel essential oil.

The FTIR spectrum of OPE, used to identify its functional groups, is presented in Figure 1. The peak observed at 3468 cm^−1^ corresponds to the stretching vibration of hydroxyl groups (–OH) in alcoholic and phenolic compounds. The peak at 2962 cm^−1^ arises from the stretching vibrations of −CH_2_ and −CH_3_ groups in alcoholic compounds (Feng 2022). Limonene, the primary component of the essential oil, exhibited characteristic bands at 1646 cm^−1^ (stretching vibration of the −C=C bond in cyclic and acyclic positions), 1440 cm^−1^ (bending vibration of the H−C bond), and 885 cm^−1^ (out‐of‐plane bending). Furthermore, the peaks at 1226 and 1050 cm^−1^ can be assigned to the rotational vibrations of −CH_2_ in alkanes or bending vibrations in H−C bonds of aromatic rings, as well as distorted vibrations of OH−C groups. Other peaks include those at 686, 960, 1100, 1444, 1750, and 1650 cm^−1^, are attributed to the vibrational absorption of alkanes, C−H bonds, C−OH and C−O bonds, C=C bonds, and C=O bonds of carbonyl groups, respectively (Ansarifar et al. 2019). The FTIR spectrum further confirms the presence of aromatic and phenolic compounds, such as D‐limonene, in OPE, as supported by studies from Cebi et al. (2020) and Noshad et al. (2021). Additionally, Feng (2022) reported the presence of other bioactive compounds, including hesperidin and naringin, in orange peel.

The total phenolic content (TPC) of OPE was determined to be 6.9 mg GAE/g (data not shown). This value is lower than that reported by Lagha‐Benamrouche and Madani (2013), who found that the TPC of various orange peel varieties ranged from 9.61 to 31.62 mg GAE/g. Similarly, Barrales et al. (2018) observed a TPC range of 1.4–15.9 mg GAE/g in OPE. These variations in phenolic content can be attributed to differences in extraction methods, solvent types and concentrations, and processing temperatures employed in their studies. Such factors significantly influence the yield and composition of phenolic compounds in essential oils.

### 3.2 Emulsion Properties

#### 3.2.1 Antioxidant Activity

Table 2 presents the antioxidant activity, zeta potential, and droplet size of microemulsions and nanoemulsions containing allicin (AM and AN) and orange peel essential oil (OPEM and OPEN). AM and AN exhibited significantly higher antioxidant activity compared to OPEM and OPEN. This can be attributed to the ability of allicin to effectively inhibit superoxide, nitric oxide (NO), and hydroxyl radicals (Nadeem et al. 2021). OPE is a rich source of phenolic compounds and demonstrates higher antioxidant activity compared to essential oils extracted from other parts of the orange (Lagha‐Benamrouche and Madani 2013). Furthermore, the antioxidant activity of nanoemulsions increased by 35.12% for OPE and 49.93% for allicin compared to their respective microemulsions. This enhancement is probably due to the increased solubility of phenolic compounds, which occurs as particle size decreases and surface area increases, allowing for greater release of bioactive compounds (Garavand et al. 2021). Lou et al. (2017) also reported that *Citrus medica* L. var. sarcodactylis nanoemulsions exhibited higher antioxidant activity than their free essential oil counterparts. According to Medeleanu et al. (2023), nanoemulsions of citrus essential oils have a larger surface area and superior solubility, which facilites the penetration of active ingredients into aqueous systems and increases their free radical scavenging capacity.

**TABLE 2:** OPE microemulsion (OPEM), OPE nanoemulsion (OPEN), allicin microemulsion (AM), and allicin nanoemulsion (AN) properties.

Emulsion type	Antioxidant activity (mg/mL)	Zeta‐potential (mV)	Droplet size (nm)	
			Day 1	Day 30
OPEM	22.19 ± 0.97	−26.50 ± 2.20	820.0 ± 34.2	1304 ± 50.8
OPEN	33.27 ± 1.20	−36.46 ± 1.90	93.5 ± 15.9	117 ± 20.1
AM	31.29 ± 0.89	−26.89 ± 2.70	640.0 ± 40.1	1070 ± 48.2
AN	42.28 ± 1.05	−36.46 ± 3.11	81.3 ± 13.3	105 ± 23.9

#### 3.2.2 The Zeta Potential and Particle Size

The zeta potential measurements revealed comparable surface charges for both formulations, with allicin microemulsions and nanoemulsions exhibiting values of −26.89 and −36.85 mV, respectively (Table 2). Similarly, OPE demonstrated nearly identical zeta potentials of −26.50 for microemulsions and −36.46 mV for nanoemulsions. Previous studies have reported comparable nanoemulsion characteristics. Lyu et al. (2024) prepared allicin nanoemulsions with a mean particle diameter of 145.27 nm and a zeta potential of −40.10 mV. Similarly, Sonu et al. (2018) characterized limonene oil nanoemulsions with slightly smaller particle size (116.60 nm) but significantly less negative surface charge (−19.64 mV).

Comparative analysis of droplet size revealed that allicin micro and nano emulsions exhibited smaller particle sizes than those of OPE. After 30 days of storage, the average particle size of OPE and allicin microemulsions increased by 59.02% and 67.18%, respectively, while the particle size of OPE and allicin nanoemulsions increased by only 25.1% and 29.15%, respectively. Therefore, both nanoemulsions demonstrated significantly higher stability compared to microemulsions, consistent with the findings of Donsì et al. (2011), who reported greater stability for D‐limonene in nanoemulsion than in microemulsion systems. Nanoemulsions demonstrate superior kinetic stability compared to microemulsions due to their nanoscale droplet size, which minimizes aggregation and gravitational separation (Mariyate and Bera 2022; Musakhanian and Osborne 2025).

In addition, no color changes, phase separation, or creaming were observed in any of the samples during the storage period. Zixiang et al. (2021) also reported a more than two‐fold increase in particle size for nano‐ and microemulsions of carnation and carboxymethyl chitosan coatings after 10 days of storage. Similarly, Lou et al. (2017) observed an increase in the particle size of *Citrus medica* L. var. sarcodactylis essential oil nanoemulsions over 30 days of storage, with no changes in visual appearance or consistency.

### 3.3 Microbial Analyses of Coated Chicken Fillets

**FIGURE 2:**
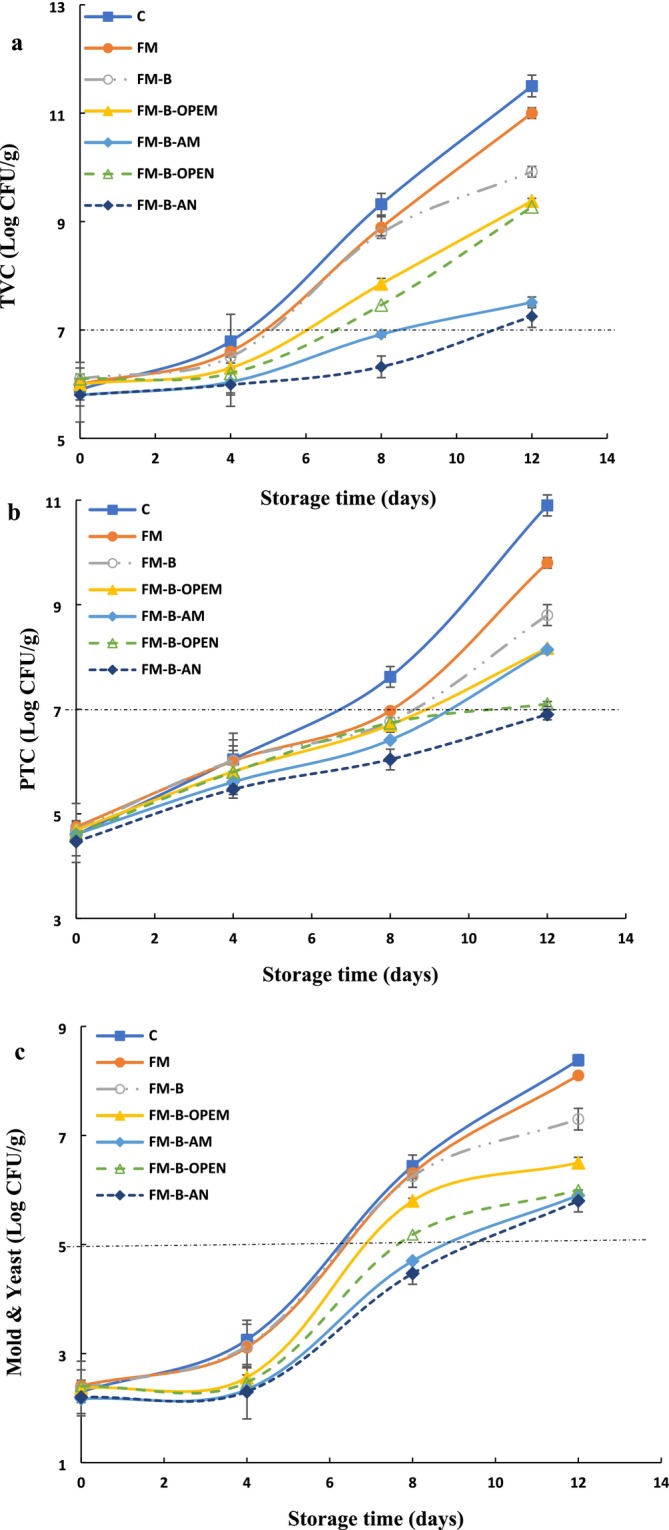
Changes in total viable mesophilic count (TVC) (a), psychrophilic (PTC) (b), and molds and yeasts (c) of chicken fillets stored at 4°C. C, Control (uncoated); FM, Flaxseed mucilage coating; FM‐B, Flaxseed mucilage + bixin; FM‐B‐AM, FM‐B + allicin microemulsion; FM‐B‐AN, FM‐B + allicin nanoemulsion; FM‐B‐OPEM, FM‐B + orange peel essential oil microemulsion; FM‐B‐OPEN, FM‐B + orange peel essential oil nanoemulsion.

The total viable bacterial count (TVC), psychrotrophic bacteria count (PTC), and mold and yeast count in different chicken fillet samples during a 12‐day storage period at 4°C are shown in Figure 2a–c, respectively. All samples exhibited a significant increase in TVC, PTC, mold, and yeast counts over time (*p* ≤ 0.05). After 12 days of storage, the control sample and the chicken fillets coated with only FM exhibited the highest growth rates for TVC (0.466 and 0.416, respectively) and PTC (0.450 and 0.420, respectively). Similarly, mold and yeast growth rates were highest in the control (0.506) and FM (0.474) samples. In contrast, the fillets coated with only FM‐B demonstrated lower growth rates for TVC (0.318), PTC (0.341), and mold and yeast (0.411). These differences can be attributed to the antimicrobial properties of bixin, which has been shown to inhibit the growth of various microorganisms, including molds, yeasts, and both Gram‐positive and Gram‐negative bacteria (Ashraf et al. 2023). Bixin interacts with the lipid bilayers of microbial cell membranes, increasing membrane permeability and causing cellular content leakage, which ultimately leads to cell death (Hosseini et al. 2024). Additionally, bixin may inhibit key enzymes essential for microbial survival and proliferation, disrupting their metabolic activities (Ashraf et al. 2023). Some studies suggest that bixin can also interfere with DNA and RNA synthesis in microorganisms, preventing replication and transcription processes and further inhibiting microbial growth (Ashraf et al. 2023; Van Chuyen et al. 2012).

Microbiological analysis revealed significantly slower growth rates for TVC, PTC, and mold/yeast counts in samples coated with FM‐B‐OPEM, FM‐B‐AM, FM‐B‐OPEN, and FM‐B‐AN, compared to control, FM, and FM‐B samples during early storage phases. OPE contains bioactive compounds such as limonene, which are known for their strong antimicrobial activity (Alparslan and Baygar 2017). Allicin is an effective antimicrobial agent that disrupts microbial cell membranes and inhibits enzyme activity. When combined with bixin, their antimicrobial effects are significantly enhanced (Choo et al. 2020; Hu et al. 2023; Savairam et al. 2023).

The most notable reduction in microbial growth rates was observed in the FM‐B‐AN sample, with growth rates of 0.120 for TVC, 0.202 for PTC, and 0.300 for mold and yeast, compared to the other samples. This demonstrates the effectiveness of the coating materials in reducing microbial growth during storage. A clear difference was observed between samples coated with nano‐essential oils and those coated with micro‐essential oils. The use of nanoemulsion coatings led to a significant reduction in microbial growth in the chicken fillet samples. Furthermore, allicin in the samples coated with FM‐B‐AN and FM‐B‐AM demonstrated greater preservation capabilities compared to OPE in the samples coated with FM‐B‐OPEM and FM‐B‐OPEN. Aerobic psychrotrophic bacteria, including *Pseudomonas*, *Shewanella*, and *Flavobacterium*, are recognized as the primary spoilage organisms in meat stored at low temperatures (Majdinasab et al. 2020; Shahrampour and Razavi 2023). The presence of allicin effectively inhibited the growth of psychrotrophic bacteria by binding to the cell walls of Gram‐negative bacteria and disrupting their cell membranes (Pabast et al. 2018).

The recommended thresholds for microbial counts indicating the onset of chicken spoilage are 7 log CFU/g for TVC and PTC, and 5 log CFU/g for mold and yeast (Shahrampour and Razavi 2023). In the control sample, the TVC reached 7 log CFU/g by the fifth day of cold storage, whereas the counts in the sample coated with FM‐B‐AN remained below this threshold even after 12 days. Similarly, the PTC in the coated chicken fillets (FM‐B‐AN and FM‐B‐OPEN) stayed below 7 log CFU/g on the 12th day, unlike the control sample (C). For mold and yeast, the control (C) and chicken fillets coated with only FM reached 5 log CFU/g after 6 days, while the counts in the FM‐B‐AN and FM‐B‐AM samples remained below this threshold until the 10th day.

While molds and yeasts can produce harmful toxins during the shelf life of food, there has been limited research on the antimicrobial effects of natural agents against these microorganisms (Duan et al. 2022). Since molds and yeasts are aerobic organisms, the reduction in their growth in coated samples is attributed to the barrier properties of the food coating, which limits oxygen penetration into the product's surface. This creates anaerobic conditions that restrict their growth in coated chicken fillet samples (Majdinasab et al. 2020). Additionally, the antimicrobial properties of allicin and OPE in the coating materials further contribute to this inhibitory effect.

The combination of bioactive compounds in OPE, such as limonene, alpha‐pinene, and beta‐pinene, creates a synergistic effect that enhances antimicrobial efficacy beyond what each component can achieve individually. Similarly, allicin demonstrates its antimicrobial properties through multiple mechanisms, including enzyme inhibition, radical scavenging, and increased permeability of microbial membrane phospholipids (Salehi et al. 2019). Allicin inhibits various enzymes by engaging in thiol‐disulfide exchange reactions with the free thiol groups of these enzymes. This interaction disrupts DNA, RNA, and protein synthesis, ultimately leading to microbial cell death (Choo et al. 2020). Other studies have reported similar findings regarding the inhibition of fungal growth during the 12‐day shelf life of chicken breast fillets. Noori et al. (2018) observed effective fungal control in fillets coated with a nanoemulsion containing 6% ginger essential oil, while Shahrampour and Razavi (2023) achieved similar results using a coating with 4% rosemary essential oil.

Findings of the current project emphasized the benefits of incorporating bixin‐loaded OPE or allicin into nanoemulsion delivery systems. These systems enhance the dispersion and targeted action of antimicrobial agents against various pathogenic and spoilage microorganisms. The reduction in particle size increases the surface area available for interaction with bacterial cell membranes, thereby improving the efficacy of these compounds as antibacterial and antiseptic agents (Hassanzadeh et al. 2023; Zixiang et al. 2021).

Hassanzadeh et al. (2023) demonstrated that coatings containing garlic extract effectively controlled TVC during the storage of mayonnaise. Their findings revealed that nanoemulsion‐based coatings exhibited superior antimicrobial effectiveness compared to microemulsions. Similar results were reported by Shahrampour and Razavi (2023), who found that chicken fillets coated with 4% rosemary essential oil maintained quality over 12 days of storage, and Hussain et al. (2021), who observed preserved quality in minced lamb containing 0.5% cinnamon bark oil after 8 days of storage. The findings of Radi et al. (2018) demonstrated that edible coatings based on OPE nanoemulsions were more effective in extending the shelf life of orange slices compared to OPE microemulsions, without affecting their sensory qualities.

**FIGURE 3:**
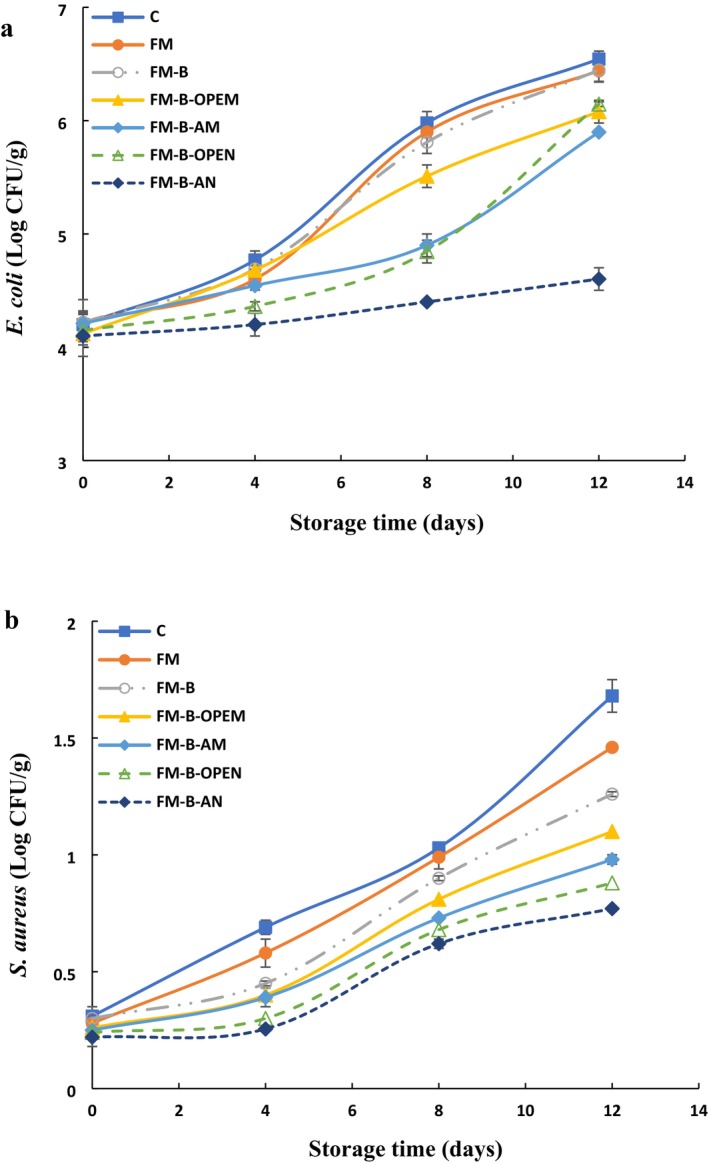
Changes in *Escherichia coli* (a) and *Staphylococcus aureus* (b) of chicken fillets stored at 4°C. C, Control (uncoated); FM, flaxseed mucilage coating; FM‐B, flaxseed mucilage + bixin; FM‐B‐AM, FM‐B + allicin microemulsion; FM‐B‐AN, FM‐B + allicin nanoemulsion; FM‐B‐OPEM, FM‐B + orange peel essential oil microemulsion; FM‐B‐OPEN, FM‐B + orange peel essential oil nanoemulsion.

The results for *E. coli* and *S. aureus* growth during storage are presented in Figure 3a,b, respectively. The initial counts for *E. coli* and *S. aureus* in all samples ranged from 4.10 to 4.21 log CFU/g and 0.22 to 0.32 log CFU/g, respectively, indicating good initial quality of the chicken fillets. However, all samples showed a significant increase in the growth of *E. coli* and *S. aureus* during storage, with the highest counts recorded in the control sample (C) 6.544 log CFU/g for *E. coli* and 1.680 log CFU/g for *S. aureus* (*p* ≤ 0.05).

In contrast, the use of flaxseed mucilage coating without essential oil (FM) was ineffective in controlling microbial growth in chicken fillets. On the other hand, the coated samples containing bixin (FM‐B) exhibited significantly lower microbial growth throughout the 12‐day storage period compared to the control (C). This reduction is likely due to the antimicrobial properties of bixin, which have been shown to inhibit the growth of various microorganisms, including *B. cereus* , *C. perfringens* , and *S. aureus* (Ashraf et al. 2023; Van Chuyen et al. 2012).

Figure 1 illustrates that incorporating essential oils into coating formulations significantly reduced the growth rates of *S. aureus* and *E. coli* compared to samples without essential oils during the 12‐day storage period. The lowest microbial counts at the end of storage were observed in samples coated with FM‐B‐AN, which recorded 5.22 log CFU/g for *E. coli* and 0.77 log CFU/g for *S. aureus* . The extent of microbial reduction was influenced by the concentration of essential oil in the microemulsion and nanoemulsion, with a more pronounced effect observed in nanoemulsion‐coated samples compared to those treated with microemulsions.

Microemulsions and nanoemulsions serve as colloidal carriers, facilitating controlled release profiles for various bioactive substances. Radi et al. (2018) further noted that these emulsion systems enhance the bioavailability of compounds with poor permeability. Nanoparticles have a unique ability to adhere to surfaces, promoting film formation. This creates a barrier effect and improves their capacity to penetrate bacterial cell walls, a process further enhanced by reducing particle size and increasing the cross‐sectional area (da Silva et al. 2023).

The interfacial tension between immiscible components in microemulsion systems is considerably lower compared to that in nanoemulsions (Fanun 2008). This reduction leads to an improved release rate of essential oils within these systems. Research has shown that nanoemulsions of carvacrol, thymol, and orange essential oils exhibit stronger antimicrobial effects against *E. coli* and *S. aureus* than their free forms (da Silva et al. 2023). Additionally, Hassanzadeh et al. (2023) reported that a 25% garlic extract nanoemulsion effectively inhibits the growth of *S. aureus* .

### 3.4 pH Changes

The effect of edible coating on pH changes in chicken fillets during storage at 4°C is shown in Table 3. The initial pH of all samples ranged from 5.85 to 5.97, within the typical range for fresh chicken meat (5.69–6.13) (Sujiwo et al. 2018). Spoilage is known to occur when the pH of chicken fillet exceeds 6.20. Over the storage period, the pH of all samples increased gradually, with the control sample (C) exhibiting a more pronounced increase. A slight pH increase was observed in coated samples; however, all values remained within the acceptable limits (pH < 6.8) throughout the 8 days of storage. Notably, these samples showed no visible spoilage indicators, in contrast to control samples and FM‐coated samples, which exhibited significant deterioration by Day 8. The pH changes of chicken fillets can be affected by several factors, including the initial pH, the composition of the coating materials, microbial activity, the balance between bacterial metabolites, including alkaline compounds and lactic acid, and the storage conditions, such as temperature and time (Majdinasab et al. 2020). Notably, samples with higher initial microbial loads exhibited faster pH increase. This pH trend aligns with microbiological counts, as volatile base production is linked to autolytic processes, which are primarily driven by microbial enzyme activity, breaking down meat proteins into alkaline nitrogenous compounds such as ammonia, amines, and trimethylamine (Choo et al. 2020; Shahrampour and Razavi 2023). Among all treatments, the control sample had the most pronounced pH change, while the smallest increase occurred in the FM‐B‐AN sample. The superior antimicrobial activity of allicin compared to OPE resulted in significantly better pH stability throughout the storage period (Çelebi et al. 2023).

**TABLE 3:** Changes in pH, hardness, weight loss, PV, and TBARs in the coated fillets chicken during storage at 4°C.

Coating Type	Storage time (days)	pH	Hardness (N)	Weight loss (%)	PV (meq O_2_/kg)	TBARs (mg MDA/kg)
C	0	5.97 ± 0.01^bA^	9.21 ± 1.12^aA^	—	1.51 ± 0.32^aD^	0.16 ± 0.001^bA^
	4	6.24 ± 0.23^bA^	6.03 ± 0.44^bB^	5.60 ± 1.02^cA^	4.81 ± 0.58^aC^	0.32 ± 0.40^bA^
	8	6.86 ± 0.11^aA^	4.94 ± 0.61^cB^	7.90 ± 0.78^bA^	8.11 ± 1.08^aB^	0.54 ± 0.35^aA^
	12	7.30 ± 0.06^aA^	3.44 ± 0.02^dB^	16.43 ± 0.71^aA^	11.72 ± 0.06^aA^	0.77 ± 0.06^aA^
FM	0	5.90 ± 0.33^bA^	9.26 ± 0.88^aA^	—	1.44 ± 1.03^aD^	0.15 ± 0.06^cA^
	4	6.20 ± 0.19^aA^	5.68 ± 0.63^bB^	5.11 ± 1.22^cbA^	4.09 ± 1.08^aC^	0.28 ± 0.15^bA^
	8	6.74 ± 0.53^aA^	4.91 ± 0.14^cB^	6.11 ± 0.55^bB^	6.03 ± 0.46^bB^	0.51 ± 0.72^abAB^
	12	7.11 ± 0.33^aA^	3.35 ± 0.18^dB^	14.10 ± 0.57^aB^	8.36 ± 0.71^aA^	0.70 ± 0.009^aA^
FM‐B	0	5.93 ± 0.56^bA^	9.59 ± 0.91^aA^	—	1.30 ± 0.09^aD^	0.14 ± 0.31^cA^
	4	6.12 ± 0.88^aA^	5.82 ± 0.71^bB^	4.29 ± 0.83^cAB^	3.87 ± 0.78^bC^	0.24 ± 0.91^bB^
	8	6.33 ± 0.37^aA^	4.97 ± 0.52^cB^	5.45 ± 0.69^bcB^	5.05 ± 0.90^cB^	0.48 ± 0.55^bB^
	12	6.96 ± 0.60^abA^	3.66 ± 0.50^cB^	10.20 ± 0.49^aC^	7.08 ± 0.32^abA^	0.56 ± 0.71^aA^
FM‐B‐OPEM	0	5.86 ± 0.90^bA^	9.57 ± 0.56^aA^	—	1.20 ± 0.58^aD^	0.13 ± 0.58^bA^
	4	6.00 ± 0.39^aA^	6.04 ± 1.72^bA^	4.71 ± 0.72^cAB^	3.36 ± 0.59^bC^	0.22 ± 0.37^bB^
	8	6.25 ± 0.83^aA^	5.18 ± 0.77^cAB^	5.30 ± 0.37^bB^	4.85 ± 0.07^dB^	0.36 ± 0.34^aB^
	12	6.81 ± 0.46^aA^	4.00 ± 0.28^dA^	8.17 ± 1.08^aD^	6.21 ± 0.58^bA^	0.45 ± 0.52^aA^
FM‐B‐AM	0	5.87 ± 0.41^bA^	9.60 ± 1.01^aA^	—	1.19 ± 0.77^aC^	0.09 ± 0.21^bA^
	4	5.98 ± 0.82^abA^	6.25 ± 0.06^bA^	4.13 ± 0.58^cB^	3.28 ± 0.99^bBC^	0.15 ± 0.72^abC^
	8	6.22 ± 0.19^aA^	5.38 ± 0.36^cA^	5.26 ± 0.82^bBC^	4.19 ± 0.09^dB^	0.20 ± 0.26^aBC^
	12	6.77 ± 0.78^aA^	4.22 ± 0.73^dA^	8.02 ± 0.27^aD^	5.19 ± 0.71^cA^	0.33 ± 0.61^aB^
FM‐B‐OPEN	0	5.90 ± 0.08^aA^	9.53 ± 0.06^aA^	—	1.17 ± 1.11^aD^	0.12 ± 0.11^bA^
	4	6.14 ± 0.59^aA^	6.14 ± 0.82^bA^	3.95 ± 2.03^cC^	3.09 ± 0.57^cC^	0.14 ± 0.16^aC^
	8	6.29 ± 0.22^aA^	5.26 ± 0.80^cA^	5.13 ± 0.16^bC^	3.97 ± 1.03^dB^	0.18 ± 0.73^aC^
	12	6.56 ± 0.76^aA^	4.28 ± 0.15^dA^	7.38 ± 0.73^aD^	5.11 ± 0.93^cA^	0.28 ± 0.44^aC^
FM‐B‐AN	0	5.89 ± 0.71^aA^	9.48 ± 0.72^aA^	—	1.15 ± 0.94^aC^	0.11 ± 0.62^bA^
	4	5.96 ± 0.06^aA^	6.32 ± 0.39^bA^	3.77 ± 0.09^cC^	3.03 ± 0.68^cB^	0.15 ± 0.23^aD^
	8	6.08 ± 0.34^aB^	5.70 ± 0.22^cA^	5.01 ± 1.03^bC^	3.93 ± 0.44^dAB^	0.17 ± 0.44^aC^
	12	6.29 ± 0.05^aB^	4.53 ± 1.31^dA^	7.17 ± 1.79^aD^	4.90 ± 0.56^cA^	0.26 ± 0.73^aC^

*Note:* Lowercase letters (a, b, c) indicate significant differences due to storage time (*p* < 0.05). Uppercase letters (A, B, C) indicate significant differences due to coating type (*p* < 0.05). Data are presented as mean ± standard deviation (*n* = 3).

Abbreviations: C, Control (uncoated); FM, flaxseed mucilage coating; FM‐B, flaxseed mucilage + bixin; FM‐B‐AM, FM‐B + allicin microemulsion; FM‐B‐AN, FM‐B + allicin nanoemulsion; FM‐B‐OPEM, FM‐B + orange peel essential oil microemulsion; FM‐B‐OPEN, FM‐B + orange peel essential oil nanoemulsion.

### 3.5 Texture Changes

Texture is a key quality parameter for assessing the freshness and overall acceptability of meat and meat products (Hashemi et al. 2017; Milani et al. 2024). As shown in Table 3, all samples initially exhibited similar hardness, ranging from 9.60 to 3.35 N. The hardness gradually decreased with prolonged storage. All treatments maintained better hardness than the control value after 12 days of cold storage, due to the preservative effects of bixin and essential oils, which helped retain initial texture while inhibiting microbial growth. Addition of flaxseed mucilage had no impact on the hardness of the coated sample (FM). Among the coated samples, chicken fillets treated with FM‐B‐AN demonstrated the highest texture retention, consistently ranging between 9.48 and 4.53 N throughout refrigerated storage. According to Li et al. (2017), the decrease in hardness of chicken fillets results from microbial protease activity, which degrades collagen and myofibrillar proteins. Allicin, however, counteracts this process through its antimicrobial properties, reducing the presence of degradative enzymes such as cathepsins and collagenases, ultimately increasing fillet hardness. Consequently, higher hardness in meat products is directly associated with lower microbial loads (Ghani et al. 2018).

During storage, samples coated with FM‐B and FM‐B‐AN exhibited a lower pH value compared to other samples, which adversely affected water‐holding capacity and preserved texture hardness. However, the application of a coating, especially when combined with bixin and essential oils, effectively minimized water loss and delayed protein degradation (Duan et al. 2022). Notably, the nanoemulsions proved to be most effective in enhancing the hardness of samples due to the smaller droplet size, which provides a larger surface area, higher free energy, and greater stability against phase separation. These characteristics lead to improved coating uniformity, ultimately enhancing texture coherence and firmness (Choo et al. 2020; Islam et al. 2023; Nadeem et al. 2021). Shaw et al. (2023) reported that polyphenols from essential oils could promote protein cross‐linking, thereby enhancing the protection of muscle proteins. Heydari et al. (2020) also observed a similar effect in ostrich meat coated with Qodume shirazi seed mucilage‐lavender essential oil.

Other studies have similarly demonstrated that coatings containing essential oils can effectively prevent the decrease in meat texture hardness. Barzegar et al. (2020) found that meat coated with *Lepidium sativum* seed mucilage containing *Heracleum lasiopetalum* essential oil exhibited increased hardness during cold storage. Conversely, Ghani et al. (2018) reported a gradual decrease in hardness over the shelf life of meat coated with a soy polysaccharide–cinnamon nanoemulsion, with the control sample showing the lowest hardness by the 8th day.

### 3.6 Weight Loss

Weight loss is a key indicator of the freshness of meat and poultry, as it can significantly affect color, taste, texture, and overall sensory quality. This weight loss occurs due to moisture evaporation from the surface of the meat products and is closely related to the storage temperature. As illustrated in Table 3, weight loss increased in all samples over the storage period, with the control samples showing the highest rate of 16.43% compared to the coated samples, which showed a lower weight change of 7.17% to 14.10%. However, no significant differences were observed between the coated samples. Notably, the FM‐B‐AN sample had the lowest weight loss of 7.17%, due to the protective barrier formed by the coating, which reduced the moisture evaporation and microbial contamination. The higher weight loss in the uncoated chicken fillet may be attributed to the denaturation of myoglobin proteins and a decreased water‐holding capacity (Mir et al. 2017; Zhang et al. 2022). Polysaccharide coatings, such as flaxseed mucilage, create a dense surface layer that acts as a barrier, retaining water within the system and resulting in minimal or no leakage (Garavito et al. 2020). In the coated sample with FM‐B, bixin can create a more effective barrier against microbial contamination, which could result in lower weight loss compared to FM and control samples.

Among coated samples, better water holding capacity was demonstrated for FM‐B‐OPEM and FM‐B‐AN samples, which had better antimicrobial activity. These coatings reduced the microbial load and minimized protein degradation, and therefore improved the preservation during extended storage (Zhang et al. 2022). The fine droplet size in microemulsions/nanoemulsions enhances emulsion stability and adhesion to the meat surfaces, creating an efficient nanoscale barrier that minimizes moisture evaporation and lipid oxidation (Mariyate and Bera 2022). Similar findings have been reported in other studies, such as fresh‐cut oranges coated with pectin–orange peel essential oil microemulsions/nanoemulsions (Radi et al. 2018) and donkey meat coated with ε‐polylysine–clove essential oil–carboxymethyl chitosan nanoemulsions (Zixiang et al. 2021).

### 3.7 Lipid Oxidation

#### 3.7.1 Peroxide Value (PV)

The peroxide value (PV) analysis of chicken fillet samples stored at 4°C over 12 days is presented in Table 3. On the first day of storage, no significant differences in PV were observed among the samples. During storage, the PV values of all samples significantly increased. However, fillets coated with FM‐B, FM‐B‐AM, and FM‐B‐AN showed significantly lower PVs compared to the control and FM samples. Bixin, a carotenoid with strong antioxidant properties, effectively scavenges free radicals and inhibits lipid oxidation (Ashraf et al. 2023). The active compounds in bixin and essential oils, when used as microemulsions and nanoemulsions, exhibit synergistic effects in combination treatments, which enhances the antioxidant activity beyond their individual contributions (Abdou et al. 2018). Orange peel essential oil contains bioactive compounds such as limonene, flavonoids, and phenolic compounds, which contribute to its antioxidant activity. According to Nurwantoro et al. (2015), the antioxidant compounds such as allicin, which is found in garlic, are comparable to synthetic antioxidants such as BHT, BHA, and tert‐butylhydroquinone (TBHQ) in inhibiting the formation of malondialdehyde.

The acceptable limit for PV in meat and poultry products is considered to be 7 meq O_2_/kg. While samples coated with OPE and allicin remained within the acceptable peroxide value limit until the sixth day of storage, both the control and flaxseed mucilage‐coated samples exceeded this threshold. However, its overall efficacy may be lower than that of allicin (Çelebi et al. 2023). Additionally, nanoemulsions, with their significantly smaller droplet size compared to microemulsions, may further enhance antioxidant efficacy. Reducing the droplet size in nanoemulsions results in a more uniform distribution within the food coating. The increased surface area of nanoemulsions enhances antioxidant properties and improves their interaction with the food matrix or oxidative species, thereby more effectively scavenging free radicals and inhibiting lipid oxidation in chicken fillets (Savairam et al. 2023).

By the ninth day of storage, all samples except FM‐B and FM‐B‐AN exceeded the standard PV limit. While flaxseed mucilage coatings reduce lipid oxidation by limiting oxygen exposure at the chicken fillet surface (Manzoor et al. 2023; Tee et al. 2016), only polyphenol‐rich compounds in bixin, OPE, and allicin significantly delayed lipid oxidation. Essential oils reduce lipid peroxidation by scavenging free radicals, inhibiting hydroperoxide decomposition chain reactions, and chelating pro‐oxidative metals (Majdinasab et al. 2020; Sheerzad et al. 2024).

Other researches demonstrate that chicken meat coated with 1.5% cinnamon essential oil in nano‐chitosan‐nanocellulose (Sheerzad et al. 2024) or 1.5% Shirazi thyme and 1% lavender nanoemulsions in basil seed gum (Majdinasab et al. 2020) exhibited the lowest PV after 12 days of refrigerated storage. Similarly, as shown in Table 2, bixin‐allicin nanoemulsion coatings displayed superior antioxidant activity, confirming allicin's strong preservative effect on chicken fat. Both allicin and OPE function as natural antioxidants, neutralizing oxygen radicals and forming a protective barrier for FM‐B‐OPEN and FM‐B‐AN samples throughout storage.

#### 3.7.2 TBARs

Changes in the TBARS values of control and coated chicken fillets are presented in Table 3. On the first day of storage, all samples showed TBARS levels below 0.2 mg MDA/kg. However, these values increased significantly over time. Among all treatments, samples FM‐B‐OPEN and FM‐B‐AN coated with Allicin and OPE nanoemulsions demonstrated the smallest increase in TBARS values during storage, indicating superior oxidative stability.

Based on TBARS values, the threshold for lipid oxidation and off‐flavor development in meat and poultry is reported to exceed 0.5 mg MDA/kg. In this study, the control sample and the sample coated with FM showed TBARS values exceeding the threshold limit by the 8th day of storage. In contrast, the coated sample with bixin reached a TBARS value of 0.56 mg MDA/kg at the end of the storage period. Other samples such as those coated with FM‐B‐OPEM, FM‐B‐AN, and FM‐B‐OPEN only reached this level after 12 days of storage. The accelerated lipid oxidation observed in the control sample is likely due to the activity of phospholipase and microbial lipases, which promote the production of free fatty acids (Pirastehfard et al. 2021). Martín‐Sánchez et al. (2014) demonstrated that annatto extract, rich in bixin, effectively reduced lipid oxidation in meat products, as evidenced by decreased peroxide values and TBARS levels, consequently improving the oxidative stability and extending shelf life. Additionally, the antimicrobial properties of allicin inhibit microbial growth and lipase production, which helps lower both peroxide value (PV) and TBARS levels in the coated samples. These findings suggest that the nanoemulsion coating effectively inhibited oxidation, with the reduced particle size of essential oils enhancing their antioxidant and oxygen‐scavenging capacities (Table 2). Zaharioudakis et al. (2023), also demonstrated that carvacrol nanoemulsion coatings were more effective than microemulsions at controlling TBARS increases in minced pork. Similarly, Hu et al. (2023) reported that allicin‐zein nanoparticle coatings in gelatin films significantly reduced TBARS levels and lipid oxidation rates in chilled beef. Barzegar et al. (2020) attributed the minimal changes of TBARS values during storage to the combined effects of the oxygen barrier properties of the coating and the antioxidant activity of phenolic compounds in essential oils. Comparable results were observed by Hussain et al. (2021), who reported a TBARS value of 1.01 mg MDA/kg in ground lamb treated with 0.05% bark oil after 16 days of storage.

## 4 Conclusion

This study successfully extended the shelf life of chicken fillets through innovative flaxseed mucilage coatings incorporating either Bixin‐loaded orange peel essential oil or Bixin‐loaded Allicin in microemulsion and nanoemulsion form. The biodegradable flaxseed coating acted as a protective barrier against moisture loss, oxidation, and microbial growth, while Bixin and Allicin provided potent antimicrobial and antioxidant effects, inhibiting pathogens like *S. aureus* and *E. coli* and preventing lipid oxidation. The microemulsion and nanoemulsion systems enhanced the solubility, stability, and bioavailability of these hydrophobic compounds, ensuring uniform distribution and increased antimicrobial efficiency (particularly with nanoemulsions) due to their high surface area. By combining bixin with allicin or orange peel essential oil as natural preservatives, this method provides a sustainable, multifunctional solution to extend the shelf life of chicken fillets while aligning with consumer preferences for clean‐label food preservation.

## Author Contributions

**Neda Hashemi:** investigation, writing – original draft, methodology, formal analysis. **Elnaz Milani:** conceptualization, investigation, writing – original draft, validation, data curation, project administration. **Arash Koocheki:** conceptualization, writing – review and editing, funding acquisition.

## Data Availability

All data generated or analyzed are included in the published article.
